# Navigating algorithm bias in AI: ensuring fairness and trust in Africa

**DOI:** 10.3389/frma.2024.1486600

**Published:** 2024-10-24

**Authors:** Notice Pasipamire, Abton Muroyiwa

**Affiliations:** ^1^Department of Library and Information Science, National University of Science and Technology, Bulawayo, Zimbabwe; ^2^Department of Languages and Arts, Nyatsime College, Chitungwiza, Zimbabwe

**Keywords:** Africa, algorithmic bias, Artificial Intelligence (AI), AI trust, information fairness

## Abstract

This article presents a perspective on the impact of algorithmic bias on information fairness and trust in artificial intelligence (AI) systems within the African context. The author's personal experiences and observations, combined with relevant literature, formed the basis of this article. The authors demonstrate why algorithm bias poses a substantial challenge in Africa, particularly regarding fairness and the integrity of AI applications. This perspective underscores the urgent need to address biases that compromise the fairness of information dissemination and undermine public trust. The authors advocate for the implementation of strategies that promote inclusivity, enhance cultural sensitivity, and actively engage local communities in the development of AI systems. By prioritizing ethical practices and transparency, stakeholders can mitigate the risks associated with bias, thereby fostering trust and ensuring equitable access to technology. Additionally, the article explores the potential consequences of inaction, including exacerbated social disparities, diminished confidence in public institutions, and economic stagnation. Ultimately, this work argues for a collaborative approach to AI that positions Africa as a leader in responsible development, ensuring that technology serves as a catalyst for sustainable development and social justice.

## Introduction

Algorithm bias significantly impacts information fairness and trust, which are vital for the successful acceptance of AI technologies (Deloitte, 2024). Recent years have seen a significant focus on bias and fairness in AI (Shams et al., 2023) as generative AI and large language models process vast amounts of data which raises concerns about privacy, discrimination, data security, and copyright infringement (Deloitte, 2024). In Africa, the deployment of AI systems has sparked critical discussions about algorithmic biases and their implications for information fairness and ethics. Key concerns include the lack of diverse datasets, implicit biases in algorithms, insufficient transparency in AI systems, and limited access to technology. Additionally, issues surrounding data privacy, ethical considerations in AI deployment, community engagement, capacity building, partnerships, and regulatory frameworks are paramount (Buolamwini and Gebru, 2018; Obermeyer et al., 2019; Jobin and Ienca, 2019).

Several definition of algorithm bias exists but they all point unfair outcomes. AI bias occurs when an algorithm's output becomes prejudiced due to false assumptions based on the data fed into it (Silberg and Manyika, 2019). According to Ferrara (2023), bias, is defined as systematic error in decision-making processes leading to unfair outcomes, is a critical concern. Ntoutsi et al. (2020) defined algorithm bias the inclination or prejudice of a decision made by an AI system which is for or against one person or group, especially in a way considered to be unfair. Algorithmic bias manifests as systematic and unfair discrimination when algorithms are employed to make decisions or disseminate information. This bias can take various forms, such as racial or gender bias, with profound consequences for individuals and communities. In the context of AI, bias can stem from diverse sources, including data collection, algorithm design, policy decisions, and human interpretation (Ferrara, 2023). Bias in AI can lead to unfair and incorrect decisions, undermining both fairness and trust. Bias mitigation is a crucial aspect in the development of fair-AI models, aimed at reducing or eliminating biases that can skew outcomes and perpetuate discrimination (Alvarez et al., 2024).

Without careful consideration of fairness and the implementation of safeguards, AI tools risk becoming instruments of discrimination, perpetuating existing injustices (Tibebu, 2024). Fairness in AI entails the absence of bias or discrimination, ensuring no favoritism is shown toward individuals or groups based on protected characteristics such as race, gender, age, or religion (Ananny and Crawford, 2016; Dwork et al., 2012). The literature proposes several types of fairness, including group fairness, individual fairness, and counterfactual fairness (Ferrara, 2023). AI and trust share an inseparable relationship (Fancher et al., 2024). Equally, trust is essential for successful human-agent interactions and significantly influences the future adoption of AI systems (Omrani et al., 2022). Trust is the expectation that digital technologies and services and the organizations providing them will protect all stakeholders' interests and uphold societal values (Dobrygowski, 2023). Rwanda's National AI Policy state that trust is critical to public confidence and acceptance of AI (Nshimiyimana, 2023).

In developing countries, algorithmic bias can exacerbate existing inequalities and impede progress toward social and economic development goals. Researchers have documented biases in AI systems against various demographics, including ethnicity, social groups, cultural backgrounds, age, and gender (Mehrabi et al., 2021; Ntoutsi et al., 2020). While AI systems themselves are not consciously biased, their decisions are influenced by the data they learn from and the algorithms they employ (Ferrer et al., 2021; Hellström et al., 2020). It is crucial to recognize that these inherent biases significantly impact information fairness and trust, particularly in developing countries.

The purpose of this article is contribute to the discourse of algorithm bias and its impact on fairness and trust with a bias toward Africa. This paper argues that addressing algorithm bias is essential for ensuring information fairness and fostering trust in AI systems in Africa. These elements are critical for successful implementation in Africa. There is a need for inclusive practices that engage local communities. Such engagement is vital for promoting equitable technological development. To this end, the following questions guided this perspective article:

- What is the current state of AI adoption and algorithmic bias in Africa?
- How does algorithmic bias impact information fairness and trust in AI systems within the African context?
- What empirical evidence illustrates the effects of algorithmic bias on fairness and trust in African AI applications?
- What strategies can mitigate algorithmic bias and promote inclusive AI development in Africa?

## Methodology

This perspective article is based on the author's personal insights and opinions, drawing from their experiences and observations related to algorithmic bias in AI systems within the African context.

### Literature review

A review of existing literature on algorithmic bias, AI fairness, and trust was conducted to contextualize the author's reflections and provide supporting evidence.

### Data sources

The author's personal experiences and observations, combined with relevant literature, formed the basis of this article.

### Limitations

This article's limitations include:

- Subjective nature of personal insights and opinions.
- Limited generalizability due to focus on African context.

## Current state of AI technology in Africa

Interest in AI has surged across the continent, driven by advancements in large language models like ChatGPT and currently, Africa is home to over 2,400 AI companies, with 40% founded in the last 5 years (Deloitte, 2024). In Africa, AI has found a wide application where it is applied in key sectors such as banking, e-commerce, health, agriculture, energy, education, and industrial manufacturing. African governments like Zambia have used AI technologies to fight electoral disinformation and misinformation; those in Libya have used AI to deploy autonomous weapon systems; in Zimbabwe, they have strengthened their surveillance systems using biometrics; and in Kenya, Ghana, and Togo, among others, the same technology has been used to develop micro-lending apps, to distribute social funds, and other initiatives (Center of Intellectual Property and Technology Law [CIPIT], 2023). Countries such as South Africa have used this technology to understand the retention of health workers in the public sector, while Kenya is home to various e-health start-ups. In Ghana, they use deep learning to automate radiology, while in Egypt, they use AI for triage and tele-nursing services. Despite the active uptake of AI systems, their use has been seen to undermine human rights and segregate marginalized groups in the society (Akello, 2022). Many African nations lack comprehensive national strategies, institutions, and regulatory frameworks to manage AI technologies effectively (Deloitte, 2024). Notable early adopters, such as Egypt, Rwanda, Ghana, Senegal, Tunisia, and Nigeria, have made strides by developing or initiating AI national strategies. For instance, Egypt launched its National AI Strategy in 2021 and established a National Council for Artificial Intelligence.

The adoption of AI technologies in Africa faces challenges, including a lack of technical skills, uncertainty, structured data, government policies, ethics, and user attitudes (Ade-Ibijola and Okonkwo, 2023). Access to digital tools is hindered by insufficient infrastructure, disproportionately affecting certain groups (Deloitte, 2024). Although internet penetration increased from 9.6% in 2010 to 33% in 2021, it remains significantly lower than in developed countries like the U.S., where it stands at 92% (Getao, 2024). A significant portion of Africa's population remains unconnected, limiting contributions to global AI models and leading to less accurate representations for local users. Due to the low internet connectivity rate, the lack of mobile phones, and the analog nature of business and transactions, critical data necessary for predictive models is lacking in Africa (Center of Intellectual Property and Technology Law [CIPIT], 2023).

Bias in AI can exacerbate existing social divisions in a continent characterized by diverse cultures and communities (Getao, 2024). The lack of a culture of sharing ideas online, rooted in historically unequal access to digital technology, complicates the situation. The National Artificial Intelligence Policy for the Republic of Rwanda recognizes the challenge of data sharing and emphasizes the importance of organizing workshops and training sessions for senior management of public departments and private companies to showcase the benefits of data sharing (Ministry of ICT Innovation, 2023). Africa is rich in data, but it has not been aggregated (Center of Intellectual Property and Technology Law [CIPIT], 2023), as many Africans primarily consume content rather than contribute to it (Getao, 2024). This creates a significant data deficit for AI development, compounded by high capital and operational costs (Deloitte, 2024). As noted by Getao, “It costs money to go online,” excluding many users from the digital landscape and increasing their vulnerability to misinformation. The high cost of mobile internet data or home-based broadband connections limits the market size and uptake of services (Center of Intellectual Property and Technology Law [CIPIT], 2023). In 2018, only 45% of Sub-Saharan Africans had mobile phones, and many devices were older models unable to support high-tech apps (Besaw and FilitZ, 2019).

Consequently, much of the data used for training AI models originates from the Global North, resulting in an overrepresentation of these populations' demographics, preferences, and behaviors (Coutts, 2024). Africa often finds itself relegated to a role of a data mine, where personal information and cultural knowledge are extracted to fuel AI models in the North (Tibebu, 2024). Unfortunately, the economic benefits generated from this data rarely return to the communities from which it was sourced, perpetuating a cycle of economic dependency and stripping Africa of agency in the burgeoning AI-powered economy (Tibebu, 2024).

### Algorithmic bias: authors' perspective on fairness and trust concerns in African context

In Africa, the concepts of fairness and trust are shaped by social and historical contexts. Historical mistrust of foreign technologies due to past exploitation influences perceptions of AI. Through mechanisms of unequal exchange, the global economy perpetuates a framework dominated by the West, siphoning Africa's wealth in the form of minerals and labor (Aseka, 1993). The question of imperialist exploitation and technological abuse increasingly occupies a central place in African discourse and algorithm bias has aggravated the issue. Senegalese expert Seydina Moussa Ndiaye warns that the biggest threat from AI is colonization, suggesting that large multinationals may impose their solutions throughout the continent, leaving little room for local innovation (UN News, 2024). Sabelo Mhlambi has called for a “decolonization” of AI (Kohnert, 2022). Democratization of AI will level the playing field in term of systems development and skills acquisition.

The perception of fairness and trust is often shaped by cultural norms, dictating what is considered equitable in various contexts. AI development and use in Africa has not been sensitive to African cultural values, beliefs, and ethical principles. For instance, the African concepts of personality contradict the notion that AI could ever be a “person”. AI systems that are viewed as impersonal or devoid of spiritual significance might be met with resistance, as people may prefer solutions that align with their cultural and spiritual values. Many African cultures emphasize social equity, community cohesion, and collective wellbeing, impacting how AI solutions are perceived. In cultures where traditional authority figures (like elders or community leaders) play a crucial role, there may be a reluctance to embrace AI technologies perceived as lacking human oversight. The philosophy of Ubuntu provides a framework for considering how AI should be developed, emphasizing that without careful handling, “through our technology and scientific developments we can easily destroy each other and the world” (Jahnke, 2021). Befittingly, Eke et al. (2023) are concerned that African values, beliefs and ethical principles are currently lacking in global discussions on AI ethics and guidelines.

Economic inequality may lead to skepticism about technologies that seem to benefit only certain groups. The Western colonial and neo-colonial interventions have fostered economic mechanisms detrimental to Africa's environment and development (Aseka, 1993). The power of AI, combined with advances in technology, could be harnessed; however, communities excluded from technological advancements may develop mistrust toward new technologies, viewing them as tools benefiting the affluent.

AI algorithms are increasingly weaponised against unsuspecting users, posing threats rather than necessities. The rise of spyware collecting personal data without consent raises significant privacy and security concerns. This misuse of AI tools can infringe on individual rights and be leveraged for illegal purposes, necessitating ethical and accountable deployment to ensure favorable outcomes. Lack of regulations can lead to distrust in data usage and management.

### Empirical evidence on algorithm bias on information fairness and trust in Africa

Empirical literature indicates the existence of biases inherent in AI algorithms, which must be addressed to avoid perpetuating discrimination and exacerbating inequalities (Shihas, 2024; Akello, 2022; Kelly and Mirpourian, 2021; Gwagwa et al., 2020; Buolamwini and Gebru, 2018). Trust cannot flourish in an environment reliant on flawed AI (Fancher et al., 2024). Notably, most algorithms are trained on biased data, compromising their effectiveness and leading to results that perpetuate discrimination. These biases stem from flawed training data, leading to discriminatory outcomes in critical sectors such as finance, healthcare, and law enforcement (Agbo, 2024). Buolamwini and Gebru (2018) examined the accuracy of commercial facial recognition APIs across genders and skin tones. Their study of 1,270 participants from Rwanda, Senegal, South Africa, Iceland, Finland, and Sweden revealed significant disparities. Dark-skinned women were misclassified at substantially higher rates than light-skinned men, who received the most accurate results. These findings highlight facial recognition technology's discriminatory outcomes, disproportionately affecting marginalized groups. However, the study's reliance on a limited dataset may underestimate the issue's true extent. This limitation underscores the need for more comprehensive research to fully capture the scope of facial recognition bias.

The integration of AI systems in various sectors poses significant risks, including personal data misuse, inaccuracies in AI outputs, and systemic biases, which can erode trust. Research has shown that algorithmic biases can perpetuate existing inequalities, particularly in financial access and hiring practices. For instance, studies have found that loan repayment prediction algorithms exhibit gender bias, resulting in lower approval rates for female borrowers (Akello, 2022; Kelly and Mirpourian, 2021; Gwagwa et al., 2020). In Kenya's fintech sector, digital lending apps rely on automated analysis of micro-behavioral data, such as browsing history and social media information, leading to biased outcomes (Akello, 2022). This disproportionately affects women with limited internet and mobile access, resulting in unfair credit scores due to inadequate digital footprints. Similarly, hiring algorithms in India have been found to discriminate against candidates from marginalized communities, perpetuating workplace exclusion (Shihas, 2024). The intersectionality of biases in AI systems compounds these issues, as overlapping forms of discrimination (race, gender, socioeconomic status) can exacerbate disadvantage.

Algorithm bias can have devastating consequences, particularly in Africa where access to digital technologies is uneven and regulatory frameworks are weak (Singh, 2022). This can lead to discriminatory outcomes, such as service denial, which undermines trust in AI technologies. For instance, predictive policing algorithms in South Africa have been found to target low-income communities, increasing surveillance and harassment of innocent individuals (Singh, 2022). Moreover, AI tools can be used to target perceived “enemies of the state,” as seen with the COMPAS software, which discriminated against African-American populations in recidivism predictions (Institut Montaigne, 2020). This highlights the urgent need for ethical guidelines and regulations to prevent potential misuse and harm. Biased algorithms can lead to unfair outcomes for marginalized communities. For instance, Obermeyer et al. (2019) revealed that a healthcare algorithm was biased against Black patients, resulting in poorer health outcomes. Similarly, studies have shown that facial recognition systems exhibit higher error rates for darker-skinned individuals (Buolamwini and Gebru, 2018).

Algorithm bias in Africa manifests significantly through the underrepresentation of diverse voices in training datasets, resulting in skewed outcomes that reinforce existing power dynamics. A striking example is the finding by Algorithm Watch Africa (2021) that recruitment algorithms often favor candidates from privileged backgrounds, perpetuating job market inequalities. Language barriers further exacerbate these challenges in Africa's diverse socio-economic landscape. The dominance of languages like English, Chinese, and French on search engines and social media limits access to information for speakers of local languages. This threatens linguistic diversity and marginalizes African voices in an increasingly AI-dependent society (Tibebu, 2024). The lack of diversity in AI datasets is a critical concern. Over-reliance on Western data leads to biases, as evidenced by Buolamwini and Gebru (2018) study on facial recognition systems. These systems exhibited higher error rates for darker-skinned individuals, with profound implications for people of African descent.

The rapid growth of AI systems has raised significant concerns about data privacy and surveillance (Deloitte, 2024). With AI's endless need for data, there's a risk of collecting vast amounts of personal and sensitive information without a clear purpose, leading to unethical and potentially illegal practices. This is particularly worrying in Africa, where governments are increasingly using AI-powered surveillance technologies to monitor citizens, often with biometric and facial recognition capabilities (Munoriyarwa and Mare, 2022). In countries like Zimbabwe, Tanzania, Angola, and Mozambique, surveillance technologies are being deployed to track citizens' activities, create profiles, and locate them (Akello, 2022). For instance, Huawei's Safe City project in Nairobi has installed 1800 HD cameras and 200 HD traffic surveillance systems, raising concerns about mass surveillance (Akello, 2022). The COVID-19 pandemic accelerated the use of surveillance technologies, with global monitoring efforts tracking the spread and severity of the disease (Center of Intellectual Property and Technology Law [CIPIT], 2023). But research has shown that AI algorithms can predict sensitive information from seemingly innocuous data (Acquisti et al., 2015). This means that even if citizens aren't actively being targeted, their personal data can still be compromised. The implications are far reaching, especially in regions with limited data protection laws.

## Discussion

Empirical literature points to the existence of biases inherent in AI algorithms, which need to be addressed to avoid perpetuating discrimination against certain groups and exacerbating existing inequalities. Transparency in AI systems is crucial, as many algorithms are complex and opaque, making it difficult for users to understand how decisions are made. This lack of transparency can lead to mistrust and skepticism about AI technologies, especially in a continent like Africa, where concerns about data privacy and security are prevalent (Ade-Ibijola and Okonkwo, 2023). Establish independent auditing bodies to assess AI systems for bias and fairness, ensuring accountability. Addressing transparency issues is key to stimulating trust and eroding skepticism among the continent. For example, the National AI Policy for Responsible AI Adoption of Rwanda emphasizes the importance of responsible AI adoption, ensuring fairness, transparency, and accountability in AI systems (Nshimiyimana, 2023).

The ethical implications of AI technologies in Africa must be carefully considered, as there are growing concerns about their use in surveillance and law enforcement, which could infringe on people's rights to privacy and freedom. Countries in Africa with weak legal frameworks risk that AI technologies could be used for authoritarian purposes, eroding democratic norms. There is need adoption of ethical frameworks in AI design that prioritize human rights and social justice.

There is an urgent need to address issues of data bias and representativeness in the development of AI technologies in Africa. Literature indicates that many AI algorithms are trained on biased datasets, perpetuating stereotypes and reinforcing existing power dynamics. To mitigate these sources of bias, various approaches have been proposed, including dataset augmentation, bias-aware algorithms, and user feedback mechanisms (Ferrara, 2023). Dataset augmentation adds diverse data to training sets to enhance representativeness and mitigate bias. Bias-aware algorithms are crafted to account for various biases and reduce their influence on system outputs. User feedback mechanisms collect input from users to identify and rectify biases within the system (Ferrara, 2023).

Furthermore, there is a need for greater diversity and inclusion in the field of AI in Africa. Sabelo Mhlambi points out that community involvement is essential in building AI systems (Jahnke, 2021). Involving local leaders and representatives in the development and implementation of AI systems to ensure they reflect community values and needs. Women and minority groups are often underrepresented in the tech industry, leading to biases in the design and implementation of AI technologies. Encouraging diverse voices and perspectives in AI development can help prevent bias and ensure that AI systems are fair and equitable for all users. As Tibebu (2024) states, empowering Africa to develop AI rooted in its cultural experiences and knowledge systems is essential for decolonising technological knowledge production. This approach offers a pathway to creating alternative narratives and perspectives that enrich the global AI landscape.

Additionally, the regulatory frameworks surrounding AI in Africa need strengthening to ensure ethical standards are upheld. Marginalized groups within Africa are particularly vulnerable to the misuse of algorithms in sensitive domains like predictive policing or social welfare allocation. Many countries in Africa lack comprehensive laws governing AI technologies, leaving them exposed to potential abuses. Policymakers must collaborate with industry stakeholders and civil society organizations to develop clear guidelines promoting fairness, transparency, and accountability in AI use. Support interdisciplinary research that examines the cultural implications of AI across different regions, particularly in Africa.

There is also an urgent need for collaboration and partnerships between African countries and international organizations. This will be key in addressing AI bias, information fairness, and ethics. African countries must prioritize the inclusion of local data, enhance digital resource access, and foster collaboration among all AI ecosystem stakeholders (Coutts, 2024). Societal progress is best achieved through collaboration and mutual support. Collaboration with cultural experts and community representatives is essential, as their insights can help ensure AI is sensitive to cultural nuances and avoids perpetuating biases (Naliaka, 2024). By sharing best practices and lessons learned, African countries can develop common standards for responsible AI use, while international organizations can provide technical assistance and capacity-building support to strengthen regulatory frameworks.

Promoting awareness and education about AI bias and ethics is essential in Africa. Many people are unaware of the potential risks and pitfalls of AI technologies, leading to unintended consequences and harms. Conduct workshops and forums to educate communities about AI technologies and their implications. Rwanda's National AI Policy note that educating the public through workshops can assist in the adoption AI (Ministry of ICT Innovation, 2023). Cultural norms surrounding education and technology literacy also play a role. By raising awareness and promoting digital literacy, policymakers can empower individuals and communities to make informed decisions about AI technologies in their daily lives. In communities where there is limited understanding of AI, misconceptions can lead to fear and mistrust.

Lastly, fostering a culture of accountability and responsibility among AI developers and practitioners is crucial. Developers, companies, and governments must ensure that AI systems are designed and used fairly and transparently (Ferrara, 2023). Create policies that mandate the inclusion of diverse cultural perspectives in AI development processes will be vital for ensuring fairness and ethics in AI use in Africa. Companies and organizations that develop and deploy AI technologies should be held accountable for any harms or biases resulting from their products. By encouraging a culture of transparency and accountability, stakeholders can work together to promote trust and confidence in AI technologies in Africa.

## Conclusions

With increased community engagement and transparency, trust in AI systems is likely to grow. As local populations see their values and needs reflected in AI applications, acceptance will rise, leading to greater utilization of technology in various sectors. By prioritizing information fairness and addressing algorithm bias, AI can contribute to more equitable economic growth. Technologies tailored to local contexts can empower underserved communities, providing access to resources, education, and opportunities that were previously out of reach. AI systems that respect and incorporate cultural norms can help preserve local traditions while also fostering innovation. For instance, AI could be used to support indigenous languages or promote traditional crafts, creating a blend of modern technology and cultural heritage. By addressing biases and ensuring inclusivity, AI can empower marginalized groups, providing them with tools to advocate for their rights and interests. AI can play a pivotal role in achieving the United Nations Sustainable Development Goals by improving access to education, healthcare, and economic opportunities.

## Recommendations

- AI developers, researchers, and policymakers should collaborate to create more inclusive and equitable algorithms
- Governments across Africa should collaborate to create a pan-African AI regulatory framework,
- Data sets used to train algorithms should be diversified to ensure underrepresented groups are included
- African languages must be considered in the design of training of algorithms
- A culture of accountability and responsibility must be fostered amongst AI developers and practitioners
- Companies and organizations that develop and deploy AI technologies should be held accountable for any harms or biases that result from their products
- Stakeholders should work together to promote trust and confidence in AI technologies in Africa, and
- African communities must be engaged to understand their information needs and preferences.

## Data Availability

The original contributions presented in the study are included in the article/supplementary material, further inquiries can be directed to the corresponding author.
